# Chronic State and Relationship to Humans Influence How Horses Decode Emotions in Human Voices: A Brain and Behavior Study

**DOI:** 10.3390/ani15213217

**Published:** 2025-11-05

**Authors:** Serenella d’Ingeo, Marcello Siniscalchi, Angelo Quaranta, Hugo Cousillas, Martine Hausberger

**Affiliations:** 1 Animal Physiology and Behavior Research Unit, Department of Veterinary Medicine, University of Bari Aldo Moro, 70121 Bari, Italy; marcello.siniscalchi@uniba.it (M.S.); angelo.quaranta@uniba.it (A.Q.); 2 Laboratoire Ethologie Animale et Humaine-EthoS, UMR 6552-CNRS, University of Rennes, 35042 Rennes Cedex, France; hugoc2@free.fr; 3 Integrative Neuroscience and Cognition Center, UMR 8002-CNRS, University of Paris-Cité, 75006 Paris, France; mhausberger.pro@gmail.com

**Keywords:** horse, emotions, physiology, animal welfare, human-animal relationship

## Abstract

**Simple Summary:**

Understanding how animals perceive human emotions is important for improving their welfare and our interactions with them. In this study, we examined how two groups of horses reacted to human voices expressing different emotions, such as happiness, anger, fear, and sadness. The horses came from very different environments: one group lived in more natural and stable social conditions, with interactions with only a few familiar humans, while the other group lived in more restricted housing and had frequent contact with many different riders. We found that horses in poorer welfare states (assessed by animal-based measures) and with less stable relationships with humans showed stronger behavioral and physiological reactions to negative emotional voices. In contrast, horses in better welfare states reacted more calmly and showed more interest in positive voices. Brain activity also reflected these differences, suggesting that both emotional state and life experience play a role in how animals perceive human emotions. These findings show that animals do not respond to emotional signals in a universal way; their individual history matters. This knowledge can help improve how we care for and interact with animals, particularly in training, handling, and welfare assessment.

**Abstract:**

Current research on acoustic encoding of emotional content suggests that there are universal cues, allowing for decoding within and across taxa. This is particularly important for human–animal relationships, wherein domestic animals are supposed to be particularly efficient in decoding human emotions. Here we investigated whether the decoding of the emotional content in human voices shared universal acoustic properties, or whether it could be influenced by experience. Emotional human voices were presented to two populations of horses, in which behavioral, cardiac, and brain responses were measured. The two populations differed in their living and working conditions: one population lived in naturalistic conditions (stable social groups in pastures) and were ridden occasionally for outdoor trail riding with one to a few different riders, while the other was kept in more restricted conditions (individual stalls) and participated in riding lessons involving many different riders. Assessment of the horses’ welfare state (animal-based measures) and their relationships with humans, performed independently of the playback experiments, revealed that the populations differed in both aspects. Whereas both populations appeared to react to the angry human voice, the population with the best welfare state and relationship with humans showed little differentiation between the different emotional voices and exhibited low behavioral reactions. On the contrary, the other population showed high behavioral and cardiac reactions to all negative voices. Brain responses also differed, with the first population showing higher responses (increased gamma, i.e., excitation) for the happy voice and the second for fear and anger (increased theta, i.e., alarm). Thus, animals’ affective state and past experiences appear very influential for their perception of (cross-taxa) acoustic emotional cues.

## 1. Introduction

Cross-taxa perception and recognition of emotional sensory cues have been an extensively studied topic over the last decade [1]. A number of playback experiments have converged to indicate that there may be some universal ability to decode other taxa’s emotions [2], although whether valence or intensity is best decoded remains under debate [3]. Indeed, emotions are usually considered as a two-dimensional phenomenon involving intensity (level of arousal) on the one hand, and valence (positive/negative affect) on the other hand [4,5]. “Classical” negative emotions would be sadness (low intensity) or fear (high intensity), positive emotions would be relaxed calmness (low intensity) or happiness (high intensity).

Thus, several authors have suggested that there are some universal features in the acoustic structures for emotion encoding [6]. The human–animal relationship constitutes an interesting model for studying this question, and there have been numerous studies on this topic on pets (e.g., dogs: [7]; cats: [8]) and other domestic animals (e.g., horses: [9], goats: [10]). Interestingly, auditory information seems to be more important than vision in determining the processing of emotional cross-modal congruence (e.g., [11]). Although there is a general agreement, based on an array of playback experiments, that domestic animals can discriminate or even recognize the emotional content of human voices (review in [12]), there is also growing evidence that this perception may be influenced by the familiarity with the voice being broadcasted (e.g., horses: [13]), the amount of exposure to humans [14], the site (e.g., goats: [15]), or the animals’ mood [16]. The results, or their interpretations, can also be unclear for unknown reasons, possibly related to the animals’ daily experience with humans (e.g., goats: [10]). Salmi et al. [17] found that captive western gorillas reacted three times more to the voices of humans associated with earlier negative experiences than to those of unfamiliar persons. Wild and domestic animals are clearly able to associate human voices with past interactions and their valence (e.g., gorillas: [17]; horses: [18]). Domestic animals are also able to both generalize their relationships with familiar humans to novel humans (e.g., horses: [19]) and modulate their responses according to familiarity (e.g., [20]). There would be no wonder, therefore, if these memories induced a bias in how individuals perceived the emotional content of unknown voices. Interestingly, negative memories of interactions tend to override positive ones [21].

Cognitive biases in the perception of sensory cues are widespread. They are well known in humans, who are more prone to preferentially process negative stimuli when suffering affective disorders such as depression or anxiety (e.g., [22]). Affective states, which reflect long term moods [4], do modulate individuals’ attention, memory processes, and judgment [23] and influence their perception of the environment [24] (reviewed in [1]). It has been demonstrated that life conditions and/or daily human actions have a large influence on the affective states of animals. Thus, improved human–animal interactions or housing conditions are associated with more “optimistic” responses in pigs [25] or rats [24], wherein animals consider ambiguous stimuli to be positive. Negative affective states, on the contrary, may be associated with anhedonia (e.g., [26]) and perception of ambiguous stimuli as negative (e.g., [24]). In a study on the human perception of conspecific and heterospecific calls with different emotional valences, Scheumann et al. [27] found that the subjects better recognized the calls with a negative valence than those with a positive valence for “familiar” species (dogs and chimpanzees); either because negative cues in acoustic structures are better conserved evolutionarily or because these calls were also better known by the public. But most of all it appeared that the human subjects were unable to recognize the emotional content of an unknown species. These results therefore contradict the hypothesis of universally shared acoustic cues encoding emotional valence, allowing cross-taxa auditory emotion recognition.

Further insight could be gained by studying the brain processes of emotions, which have been well studied in humans using non-invasive electrophysiological (electroencephalography: EEG) surface recordings. These studies have revealed correlations between the types of waves produced and the internal state. For example, relationships have been found between brain electrophysiological and neuroendocrine activities in regard to affective state (e.g., [28]), Indeed, electroencephalography is a useful tool for identifying the valence of a behavioral response through lateralized brain activity and its intensity through the wave types produced. Different wave types have been identified from delta waves (0–3 Hz), characteristic mostly of deep sleep, to gamma waves (>30 Hz) associated with higher arousal levels, and theta (3–8 Hz, low arousal), alpha (8–12 Hz, active awake), and beta (12–30 Hz, moderate arousal) waves being intermediate [29,30]. Analysis of the proportions of the different wave types (quantitative EEG power spectrum) is considered a valid tool for assessing the link between brain processes and affective or cognitive states in humans [30]. EEG power spectrum analysis thus allows us to identify both (1) the emotional valence given to a stimulus by studying the differential activation of the right versus left hemisphere (e.g., [31]) and (2) its emotional arousal by investigating wave frequency band proportions produced in response to the given stimulus [32]. For example, an increased proportion of theta oscillations is observed in memory encoding, retrieval, and emotional regulation in both humans and horses [33,34] whereas gamma waves are linked to heightened attention related to emotional arousal, including emotion processing and sensory integration [29,35]. Interestingly, theta and gamma oscillations often show opposite modulations, suggesting distinct neural processing patterns [18]. Actually, different studies describe two types of theta waves, with one demonstrating an involvement either in “conscious quiet awakeness” and “bliss” [36] related to movements, or, in the other case, in response to negative emotional stimuli in humans or threats, inducing “freezing” (immobility) (e.g., in rats facing a cat) (e.g., [37]). Functional asymmetries provide further insight into emotional and attentional processing, as observed in human EEG studies, which reveal the left hemisphere’s association with positive emotions and the right hemisphere’s involvement in negative or alarm states (e.g., [31]) or animal studies that show a link between the right hemisphere and increased attention [38].

Therefore, in the present study, we tested the behavioral responses of 27 adult domestic horses, and the cardiac and EEG responses of a subset of horses, to the playback of standardized (i.e., unfamiliar) non-verbal human voices bearing four types of emotions of varied valence and intensity: positive and high (happiness), negative and low (sadness), and negative and high (fear and anger) [7]. In order to disentangle the role of possible universal features from the influence of affective states and earlier interactions with humans, we tested horses from two populations living in opposite conditions (naturalistic setting with group outdoor housing and relaxed interactions with a few humans—14 non-restricted condition horses; restricted conditions with single stall housing, limited access to hay, and more tense interactions with a large number and variety of humans—13 riding school horses). An earlier study on horses from two such populations has revealed that they also had opposite affective states [39] and reacted differently to familiar voices associated with different valences of past interactions [18]. Moreover, in order to better evaluate the role of affective states and relationships with humans on the individual horses’ responses to human emotional voices, independent assessments of their welfare states through direct observations and of the horses’ relationships with humans through standardized tests were performed. A welfare assessment was made through animal-based measures, in accordance with Lesimple [40] and the following definition: “a chronic positive mental and physical state resulting from the satisfaction of the animal’s behavioral and physiological needs and expectations. This state can vary according to the perception of the situation by the animal” (e.g., [41]).

During playback experiments, voices lasted 1 s, which is well within the range wherein the brain can cognitively process emotional stimuli [42,43], and were all female voices, as in both populations of horses, the people they regularly encountered included a large majority of women. The loudspeaker was placed behind the horses (in order to check for possible lateralized head turns) at 10 m and the behavioral, cardiac, and brain responses were recorded. Horses were slightly restrained by a handler during the auditory tests. Thanks to the development of a telemetric system of EEG recording for horses [44], earlier studies have shown that EEG power spectrum profiles (i.e., proportion of the different types of waves: slow: alpha, theta, and fast: gamma, beta) of horses were influenced, in resting EEG, by welfare state and, in response to the playback of visual or auditory stimuli, by attention and affective state [18,45,46]. Playback studies using species-specific or human voices have shown a lateralization of horses’ responses which varies according to the stimulus valence or interest but also involves a two-step processing, initial perception, and then cognitive analysis (e.g., different ear–head latencies or changes between immediate and subsequent laterality [18,47]. Thus, in d’Ingeo et al. [18] the initial positive perception (left hemisphere-dominant) was followed by a right-hemisphere engagement (related to heightened attentional and memory processes). Finally, cardiac responses were also recorded through a telemetric device [7] but could only be recorded for the stabled horses, as the outdoor horses had still a thick winter fur at the time of the study.

## 2. Materials and Methods

### 2.1. Participants

The experiment involved 27 horses of various breeds (17 Unregistered; 2 Thoroughbred; 4 French Pony; 1 French saddlebred; 1 Angloarab; and 2 mixed breed), aged between 2 and 22 years (10.48 ± 5.58; mean ± s.d.) (Table S1). As in d’Ingeo et al. [18], participants belonged to two populations which differed in their living/working conditions. Briefly, Non-Restricted Conditions horses (NRC) (N = 14:3 stallions, 6 mares, 5 geldings, aged 2 to 22 years; 9.71 ± 7.15; mean ± s.d.), belonged to the University of Rennes or to a private owner, and lived in naturalistic conditions: in 1–2 ha pastures within stable social groups. Interactions with humans were mostly restricted to food and water when needed, but they had all at least been trained for routine care using positive reinforcement, and they were occasionally used for track riding in the open (i.e., low hands and reins held loose). Restricted Conditions horses (RC) (N = 13: 7 mares, 6 geldings, aged 5 to 17 years (11.31 ± 3.25; mean ± s.d.) belonged to a riding center and lived in restricted conditions: kept in single stalls (3.4 × 3.3 m) and with limited access to hay (provided twice per day). They worked in riding lessons from beginners to advanced stages with constrained riding techniques (English riding style, see [46]) for 3–4 h per day. All horses had been in their respective facilities, and under the same management practices, for at least one year.

### 2.2. Emotional Voices

Horses were presented with emotional human voices displaying happiness (laughs), fear (screams), anger (growls), and sadness (sobs). The stimuli used were taken from Siniscalchi and colleagues’ study on dogs [7], see examples in Figure S1. These voices were recorded from 14 human volunteers and validated by a separate sample of volunteers (for a detailed description, see [7]). Since both NRC and RC horses interacted the most frequently with women, we decided to present only female acoustic stimuli to the animals. A total of eight voices (two × each emotion) were selected so that two sets of four emotional acoustic stimuli were used. Horses were randomly assigned to one of the two sets and the number of individuals presented with the same set of stimuli was balanced between the two populations. The playback order of each set was randomized between subjects. Acoustic stimuli had a mean duration of 1 s. The stimuli were equalized, and their amplitude was homogenized to obtain an average loudness of 60 dB when measured from the horses’ head position (evaluated by PHONIC PAA 3).

### 2.3. Testing Procedure

**Figure 1:**
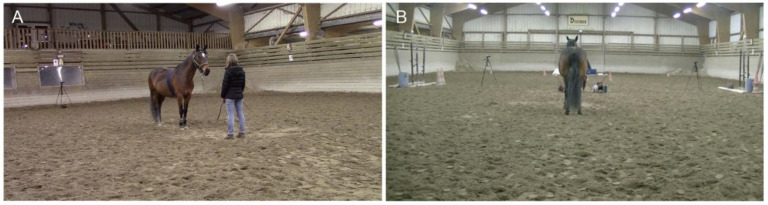
Representation of the experimental set-up: (**A**) view from one of the lateral cameras; (**B**) view from the back camera.

The experiment was carried out in the horses’ living environment in order to avoid any potential influences of the place novelty on the animals’ stress and vigilance levels. Horses were tested individually, either in a familiar outdoor or covered arena for the Non-Restricted Conditions horses (NRC) or in the riding center’s covered arena for the RC population. All horses were used to being handled separately and led to these arenas for training or riding lessons, respectively. Emotional human voices were broadcast by a loudspeaker (MIPRO MA-101C, MIPRO Electronics Co., Ltd., Taiwan, China) placed centrally behind the tested animal, at a distance of 10 m (see also [18]). It was connected to a computer located in front of the horse at a distance of 2 m. An experimenter (S.d.) controlled the stimulus broadcasting and the EEG recordings from the computer. She was positioned centrally and was asked not to interact with the tested animal, avoiding biasing horses’ lateralized response and behavior. Three video cameras (CANON LEGERIA HRF 806, CANON, Tokyo, Japan and JVC GZ-RX645BE, JVC, Yokohama, Japan) were used to record the animals’ responses to the emotional human voices. They were positioned on a tripod and placed centrally behind the animal and at its two sides (Figure 1).

### 2.4. Procedure

**Figure 2:**
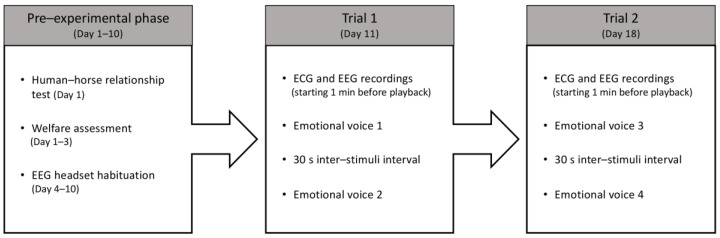
Schematic representation of the experimental protocol.

The test consisted of two weekly trials in which two different emotional human voices were presented to each horse individually (Figure 2). In each trial, two different acoustic stimuli were played. Three unfamiliar experimenters (from the horse’s perspective) alternately handled the horses during the experiment. Each experimenter stood centrally in front of the subject to avoid introducing bias in the horse’s lateralized responses. The horses were held on a loose lead rope, allowing them to move their head and neck and to step forward/backward or turn at will. Prior to broadcasting the stimuli, once the horse reached the predetermined position (i.e., facing the experimenter and symmetrically aligned with the loudspeaker), a 1 min recording of cardiac and brain activity was initiated. This recording was later used to establish a baseline for cardiac activity. Afterward, once the horse’s head was centrally positioned (to avoid bias in the head-turning response), the voices were played, with a 30 s interval between each. The horses were halter-restrained for 15 s. Throughout the test, the experimenters remained motionless, silent, and avoided interaction with the subject by facing the ground, so that horses had no access to visual interaction with or facial expressions from the handler. The emotional voices were presented to the horses in a pseudo-randomized order.

### 2.5. Data Analysis

#### 2.5.1. Behavioral Analysis

The horses’ behavioral responses to emotional human voices were video recorded during the tests. The interval between stimulus onset and the head-turning response (i.e., latency) and the direction of the turn (i.e., right or left) were examined, with both measurements performed within a 6 s threshold from the voice onset. This threshold was established through visual examination of the frequency distribution of head-turning responses. It was set based on the observed significant decline in responses occurring after 6 s from stimulus onset, following the method described by d’Ingeo and colleagues [18]. Head-turning responses occurring within 6 s of the sound onset were considered for analysis: right turn (scored as −1), left turn (scored as −1), and no response (if the horse did not turn its head within 6 s of sound onset, scored as 0). Horses’ behavior was analyzed throughout the test and classified into three behavioral categories: vigilance/alarm (standing still, body tension, head/neck up, eyes open and alert, tail raised, dilatated nostrils), frustration (vacuum chewing, lip movements, head shaking, pawing, yawning, scratching, go away), visual attention directed toward the loudspeaker (glance at the loudspeaker < 1 s, gaze at the loudspeaker > 1 s, head and eyes directed toward the loudspeaker, body turning toward the loudspeaker) [48] (see Table S2 for detailed description). The frequency of each behavior during the test was calculated. Moreover, the time spent gazing at the loudspeaker was computed for each emotion. The time spent with different ear positions (forward, backward, and asymmetric, see [21]) was also explored using the classical ethological “instantaneous scan sampling” (i.e., it was registered as a scan every 2 s) [49]. Video recordings of the test sessions were analyzed frame by frame by two trained observers. Inter-rater reliability was assessed through independent parallel coding of the horses’ behavior and calculated as a percentage agreement. For all behavioral variables assessed, inter-rater agreement exceeded 95%.

#### 2.5.2. Electroencephalography and Electrocardiography

**Figure 3:**
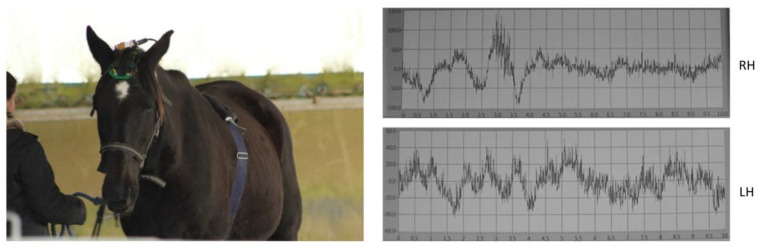
EEG headset and ECG device used in the study (**left**). Representative 10 s EEG recording acquired using the EEG device (**right**). RH: Right Hemisphere. LH: Left Hemisphere.

**Figure 4:**
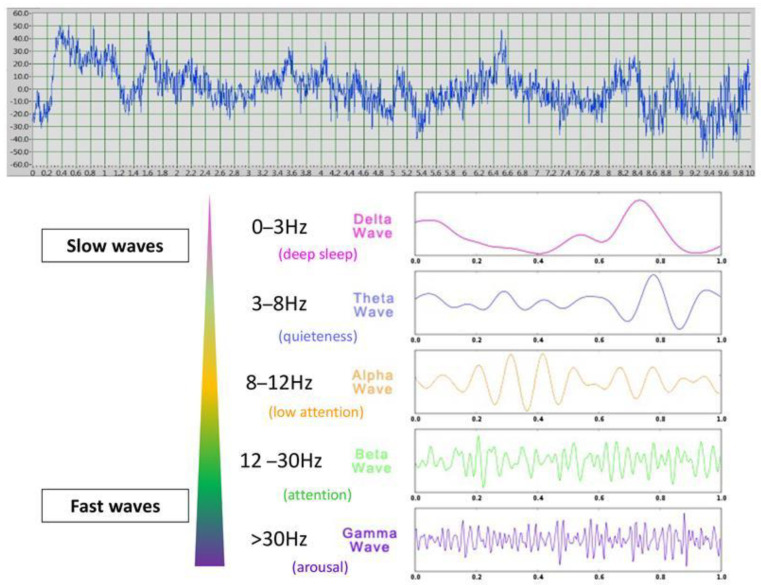
An example of 10 s EEG recording and the different types of waves: fast waves (beta and especially gamma waves are classically associated with higher levels of attention/vigilance/arousal) whereas slow waves (alpha and theta are associated with lower levels of attention).

The horses’ cardiac activity was registered employing the PC-Vetgard + tm Multiparameter wireless system for telemetric measurements (VMed Technology, Mill Creek, WA, USA), previously used by Siniscalchi et al. [7] (Figure 3). The device was made up of three integrated electrodes and a wireless ECG data-transmitting unit kept in contact with the animal’s chest by an elastic surcingle. No habituation to the ECG device was necessary, as the subjects were already accustomed to wearing girths during regular riding activities. The cardiac activity registered before the stimulus playback (1 min before broadcast) in each trial and in response to the acoustic stimuli was used to obtain the heart rate (HR) baseline and the heart rate curve, respectively. The cardiac activity recordings were analyzed only for the horses belonging to the riding school, since the other group of animals still had a thick coat that hindered an adequate contact of electrodes with the chest, which is necessary for an appropriate recording. The heart rate (HR) curve was segmented by a baseline, dividing it into two components: the Area Under the Curve (AUC), representing the portion of the HR curve above the baseline, and the Area Above the Curve (AAC), representing the portion below the baseline. Both areas were calculated to assess cardiac reactivity. Specifically, the AUC and AAC values, which included HR values higher and lower than the baseline, respectively, were computed as a number of pixels using Adobe Photoshop^®^. Heart rate changes in response to the different emotional voices were then analyzed by comparing the area values obtained [7]. Horses’ brain activity was registered using a non-invasive EEG headset developed by Cousillas et al. [44] (patent # R23701WO) that has been recently employed in several studies [18,45,46,50]. Briefly, it was composed of a telemetric EEG recorder and an amplifier connected to a Bluetooth transmitter, which allowed the recording of unrestrained animals. The device was made up of four electrodes placed on the two sides of the horse’s parietal and frontal bones (two for each side) and of a ground electrode positioned behind the left ear (Figure 3). Horses grew accustomed to the headset during daily visits in their living environment for a week before the experimental tests. During the visit, the device was placed on the horses’ heads for about half an hour until no stress-related behaviors were displayed. Since the stimuli were 1 s long and horses’ first significant behavioral reactions to sounds are within 2 s [47], EEG spectral power change was measured considering the 2 s before the stimuli onset (baseline) and 2 s after the stimuli onset to detect any change in the horses’ EEG profile (proportion of the different waves) that could reflect the cognitive aspect of emotional processing. Specifically, the median of the percentage values of delta (δ: 0–4 Hz), theta (θ: 4–8 Hz), alpha (α: 8–12 Hz), beta (β: 12–30 Hz), and gamma (γ: >30 Hz) waves for each emotional voice were computed as in d’Ingeo et al. [18] (Figure 4). Before the analysis, large artifacts related to body movements were removed, employing a smooth Savitzy Golay function integrated in a software working in the Python 3.6.4 environment. The device sampling rate was 250 Hz and the electric signal was registered using an EEG software developed by RF-TRACK [18,44,45,50]. After the visual removal of large artifacts and precise analyses of data, only horses with complete EEG recordings from both the right and left hemispheres, and for all emotional stimuli presented, were included in the analysis (5 NRC and 6 RC horses). There was no difference between populations in the morphological characteristics of these horses: there were, respectively, 3 and 3 individuals of “horse type” (>1.45 m at withers) and 2 and 3 of “pony type” (<1.45 m at withers) with similar head shapes in both sites (Figure S2). This also ensured that differences between populations could not be related to head morphological differences. The physiological responses of the animals to emotional voices were recorded as follows: heart rate was measured for 10 s from the onset of each playback, and brain activity was analyzed during the first 2 s following stimulus onset (as detailed in the rationale above).

#### 2.5.3. Welfare Assessment and Human Animal Relationship Tests

Animal welfare and the horses’ relationships with humans were assessed employing the validated tests and related methodology described in Stomp et al. [45]. Briefly, in the home stall, the percentage of scans with ears in a backward position while feeding on hay, the number of abnormal repetitive/stereotypic behaviors, and horses’ aggressive behaviors toward humans during standardized tests were used as indicators of compromised welfare. Ear positions were recorded during 30 min while the horses were feeding on grass (NRC horses) or hay on the ground (RC horses), both in the morning and in the afternoon of two consecutive days. Instantaneous scan sampling with a 2 s scan were employed for the analysis, and the number of scans for each ear position (i.e., forwards, backwards, sideward and asymmetrical) were computed. The percentage of scans of ears in a backward position was then calculated for each horse. The number of stereotypic behaviors (per hour) was scored while the horses were in the home stalls/pasture. Observations were performed three times per day (60 min per each horse) in the morning (10:00–12:00 a.m.), afternoon (2:00–5:00 p.m.), and 30 min before meals (9:30 a.m. and 6:00 p.m.). The horses’ behavior toward humans was observed during three standardized human–horse relationship tests (HHRT), presented in the following order: a sudden approach test, repeated five times (e.g., [19]), where an experimenter suddenly appeared from the closed door of the box while the animals were feeding; a motionless person test, where the same experimenter entered the box and remained motionless for 1 min; and an approach–contact test (e.g., [21]), where the experimenter approached the animals perpendicularly from a distance of 1.5 m, one step per second, up to the neck level. The number of agonistic behaviors (ears laid back, threats and attempts to bite) and positive behaviors (approaching with ears forwards, sniffing, nibbling) were registered.

On the basis of the behavioral and postural data, a chronic stress score (TCSS) that reflects how much the chronic welfare state is altered, as in 40 and adapted from Hausberger et al. [51]’ and Henry et al. [39]’s studies, was calculated for each horse. TCSS calculation consisted of ranking the horses according to (1) their number of aggressive responses during the three human–horse relationship tests, (2) the number of stereotypic behaviors displayed in 60 min of all-occurrence sampling sessions, (3) the percentage of scans spent with ears backwards while feeding. For all of these variables, the higher the value obtained was, the poorer the welfare state was and the higher the rank attributed to the horse was. Ranks were added up between variables for each horse, such that at the end, the poorer the welfare of the horse, the higher its TCSS. For instance, a horse that ranked 9th lowest according to its number of aggressive responses, 8th lowest according to its number of stereotypic behavior occurrences, and 5th lowest according to its percentage of scans spent with ears backwards while feeding, achieved a TCSS of 22, which was higher, and consequently reflected poorer welfare, than a TCSS of 3 obtained by a horse ranked 1st lowest in all these variables. Three composite scores were calculated according to the study population considered: one with all subjects combined (TCSS 1, N = 27), and one for each of the two populations, calculated by separately ranking the horses belonging to the Restricted Conditions and Non-Restricted Conditions populations. The welfare assessment and evaluation of the human–animal relationships of the horses were conducted by a different experimenter from the one performing the playback experiment (both experimenters were blind to the results of the other part of the experiment). This experimenter was not familiar with the horses, and the assessment was conducted one week prior to the playback experiment (Figure 2).

### 2.6. Statistical Analyses

Statistics were performed with SPSS software version 22. Data distribution was assessed by the Shapiro–Wilk test. A generalized linear mixed model (GLMM) was applied to all behavioral parameters (head turning, vigilance/alarm, frustration, visual attention toward the loudspeaker, ear position, and time spent gazing at the loudspeaker) to evaluate the effects of group (RC vs. NRC), emotion (happiness, sadness, anger, fear), and their interaction (group × emotion), with subjects included as a random factor. As no significant interaction was detected, and given the small EEG sample size which prevented GLMM use for EEG data [52], we proceeded with separate analyses comparing emotions and groups independently.

Binomial tests were used to determine whether head-turning behavior occurred at a frequency different from chance (50% probability). Friedman tests were conducted to assess differences between emotions for each behavioral parameter. Post hoc analyses, including Wilcoxon signed-rank tests or paired-samples *t*-tests (based on data distribution), were used to explore differences within behavioral parameters. Bonferroni correction was applied to adjust *p*-values for multiple comparisons.

Wilcoxon signed-rank tests were also applied to detect differences in heart rate, specifically comparing AAC and AUC in RC horses. Head-turning asymmetries were assessed using a one-sample Wilcoxon signed-rank test to identify significant deviations from the hypothesized median of zero.

Differences between Non-Restricted Conditions (NRC) and Restricted Conditions (RC) horses in their behavioral responses to emotional voices (including head turning, vigilance/alarm, frustration, visual attention, ear position, and time spent gazing at the loudspeaker) were tested using Mann–Whitney U tests or independent samples *t*-tests, depending on data distribution.

Mann–Whitney U tests were also used to compare NRC and RC horses regarding the relationship between welfare state (expressed by the Total Chronic Stress Score, TCSS) and their interactions with humans (expressed by the number of positive and negative behaviors during the Human–Horse Relationship Test, HHRT).

Additionally, Spearman correlations were performed to explore relationships between TCSS, positive and negative behaviors during the HHRT, stereotypic behaviors, and the analyzed behavioral parameters (latency, horse behavior, ear position, time spent gazing at the loudspeaker) as well as heart rate changes.

For brain activity, Sign tests and Wilcoxon signed-rank tests were used to analyze differences in the median percentage of different wave frequencies between baseline and the responses to emotional acoustic stimuli in each hemisphere for both RC and NRC horses.

## 3. Results

### 3.1. Horses Show Differentiation of Negative Versus Positive Emotional Cues in Human Voices at the Overall Population Level

**Figure 5:**
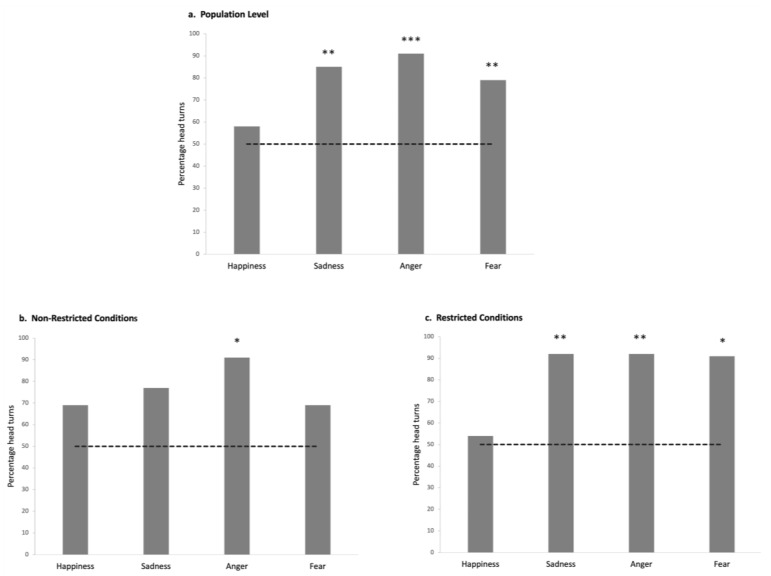
Proportion of horses that turned their heads after the playback of each emotional voice: (**a**) at the overall population level; (**b**) in the Non-Restricted Conditions population; (**c**) in the Restricted living conditions population. Chance line represented by a dotted line. Binomial test. *: *p* < 0.05; **: *p* < 0.01; ***: *p* < 0.001.

The GLMM analyses revealed no significant interaction effects of group, emotions, and group × emotion on the behaviors studied (all *p* > 0.05). At the overall population level, more horses than expected by chance turned their heads toward the loudspeaker in response to the negative emotional voices (sadness: N= 26, *p* = 0.001; anger: N = 23, *p* = 0.000; fear: N = 24, *p* = 0.007; Binomial test) but not to the happy voice (N = 15; *p* > 0.05; Binomial test; see Supplementary Information for mean and standard deviation for each variable analyzed; Tables S3–S6) (Figure 5a). However, no significant differences were found in the latency of the reactions (N = 19; *p* > 0.05; Friedman test) nor in terms of laterality (of head turning) according to the voice type (*p* > 0.05; One-Sample Wilcoxon Signed Ranks Test). Only one other behavior varied statistically according to the type of voice: horses spent more time with their ears backwards when angry voices were broadcasted (N = 25; Friedman test: χ^2^(2) = 12.02; *p* = 0.002; pairwise comparisons: backward vs. forward: N = 26; Z = 203.00; *p* = 0.036; backward vs. asymmetric: N = 25; Z = 303.00; *p* = 0.000; Wilcoxon signed rank test). No other statistically significant differences were observed for the behaviors analyzed across the different emotional stimuli (*p* > 0.05).

Therefore, at the overall population level, horses only turned their heads towards the loudspeaker for the negative voices and also showed a slightly more negative reaction to the angry voice (ears kept backwards).

### 3.2. Horses from Two Different Populations React Differently to Emotional Human Voices

**Figure 6:**
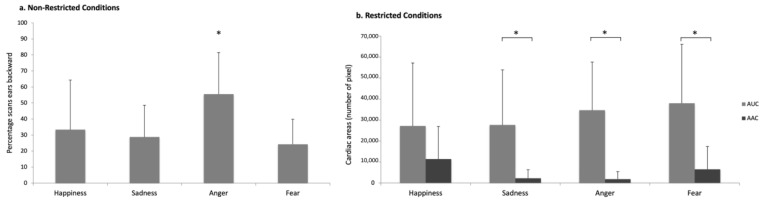
Behavioral and cardiac response to the emotional voices in the different populations: (**a**) percentage of scans with the ears backwards in Non-Restricted Conditions; (**b**) cardiac activity represented by the Areas Under Curve (AUC) and Above Curve (AAC) in Restricted Conditions horses. Means with s.d. are shown. Wilcoxon signed rank test. *: *p* < 0.05.

More Non-Restricted Conditions horses (NRC) significantly turned their heads in response to the angry voice (N = 11, *p* = 0.012; Binomial test) than expected by chance, but this was not the case for any of the other voices: *p* > 0.05, Binomial test (Figure 5b). They also spent more time with their ears backwards (N = 11; χ^2^(3) = 9.61; *p* = 0.022, Friedman test) after the playback of anger than after that of the other negative emotions (sadness: N = 12, Z = 60.00; *p* = 0.045; fear: N = 12, Z = 3.5; *p* = 0.036, Wilcoxon signed rank test) (Figure 6a). No laterality bias in head turning was observed, with the exception of significant right head-turning in response to fearful voices (N = 13, Z = 20.0, *p* = 0.020, One-sample Wilcoxon signed rank test). No other behavior showed statistically significant differences across the different emotional stimuli (*p* > 0.05).

The Restricted Conditions horses (RC) appeared especially reactive to negative voices: significantly more of them (than expected by chance) turned their head for all the negative (but not positive) voices (sadness: N = 13, *p* = 0.003; anger: N = 12, *p* = 0.006; fear: N = 11, *p* = 0.012; happiness: N = 7; *p* > 0.05; Binomial test, test proportion: 0.5; Figure 5c). Negative voices also elicited a significant increase in RC horses’ cardiac activity with respect to the baseline (AUC values vs. AAC: sadness: N = 7; Z = 1.00; *p* = 0.028; anger: N = 7; Z = 0.00; *p* = 0.018; fear: N = 11; Z = 6.00; *p* = 0.016, Wilcoxon signed rank test) (Figure 6b) whereas no differences were found for happiness (N = 9; *p* > 0.05; Wilcoxon signed rank test). No other statistically significant differences were observed for the behaviors analyzed across the different emotional stimuli (*p* > 0.05).

Finally, the comparison between Restricted Conditions horses (RC) and Non-Restricted Conditions horses (NRC) revealed significant differences in their reactions to the different emotional voices, particularly for the voices with a negative valence: RC horses were more attentive toward the loudspeaker (N = 24; t = −2.57; *p* = 0.018, *t*-test independent sample) but also spent more time with their ears backwards for the fearful (N = 23; t = −3.42; *p* = 0. 003; *t*-test independent sample) and sad (N = 25; t = −2.61; *p* = 0.017, *t*-test independent sample) voices than NRC horses. They showed more vigilance/alarm behaviors (N = 25; U = 121.00; *p* = 0.019; Mann–Whitney test) and attention (N = 24; U = 106.00; *p* = 0.047, Mann–Whitney test) after the playback of the sad voice than the other group. RC horses reacted faster to angry voices than NRC horses (N = 23, U = 38.00, *p* = 0.05, Mann–Whitney test). No other statistically significant differences were observed between the two horse groups for the behaviors analyzed across the different emotional stimuli (*p* > 0.05).

Therefore, NRC horses showed little differentiation between the emotional voices but reacted slightly more negatively to the angry voice and turned their head to the right (left hemisphere) more frequently for the fearful voice. RC horses, on the contrary, reacted strongly to all negative emotional voices, as confirmed by their cardiac response after the playback of these voices. RC horses clearly differed from NRC horses in the amount and type of behavioral reactions to the playback of each negative emotional voice, showing much more arousal and alarm or discomfort.

### 3.3. Perception of Emotional Voices Is Reflected in Brain Activity

**Figure 7:**
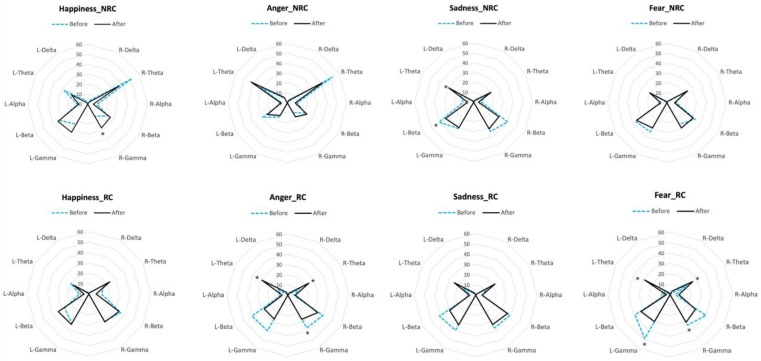
Differences in the EEG power profiles for each population (NRC: Non-Restricted Conditions horses; RC: Restricted Conditions horses) before the stimulus onset (dotted lines) and in response to the emotional voices (after the stimulus onset; black lines). Each radius represents the median proportion of each wave’s frequency in each hemisphere (R: right hemisphere; L: left hemisphere). Sign tests and Wilcoxon signed-rank tests. *: *p* < 0.05.

We found significant differences between the baseline and the brain response to the emotional voices in the RC and NRC populations (Figure 7), but again, the EEG profiles of both populations differed. In the NRC population, we observed an increase in gamma waves in the right hemisphere in response to the happy voice (N = 5; *p* = 0.031, Sign Test). In response to the sad voice, there was a decrease in beta waves and an increase in theta waves in the left hemisphere (beta decrease: N = 5, *p* = 0.031 in both cases Sign Test). No other significant changes were observed. In RC horses, there were no changes in the EEG profile for happiness but both fearful and angry voices elicited a bilateral increase in theta waves (fear: N = 6; Z = 2.2, *p* = 0.028; anger: N = 6; Z = −2.02, *p* = 0.028, for both left and right hemisphere, Wilcoxon signed rank test) associated with a decrease in gamma waves in both (fear: left: N = 6; Z = 2.02, *p* = 0.027; right: N = 6; Z = −2.02, *p* = 0.028; Wilcoxon signed rank test) or the right (anger: N = 6; Z = −2.02, *p* = 0.028; Wilcoxon signed rank test) hemispheres. Finally, sad voices elicited a significant decrease in beta activity in the left hemisphere (N = 5, *p* = 0.031; Sign Test). EEG recordings confirm the cognitive bias of RC horses towards the negative emotional voices with a bilateral increase in theta waves in response to the fearful and angry voices and no change for the happy voice. On the contrary, NRC horses showed arousal (gamma waves) for the happy voice and no change for fear or anger. Both populations showed a decrease in beta waves in the left hemisphere for sadness, though.

### 3.4. Individual and Population Responses to Emotional Human Voices Are Influenced by Horses’ Welfare State

**Figure 8:**
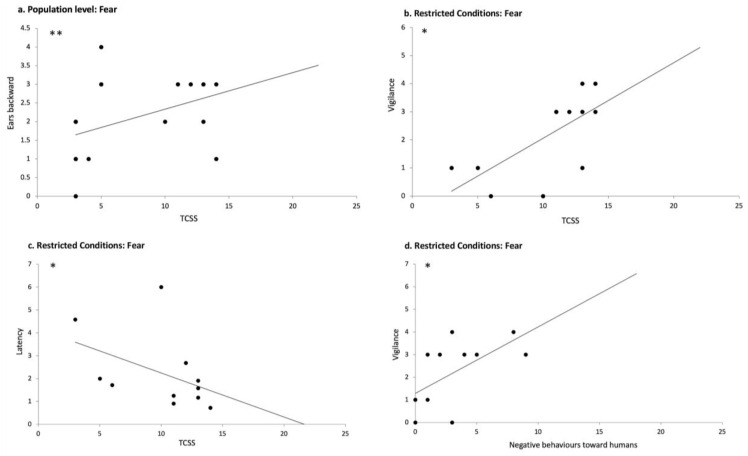
Correlations between welfare state, the relationship to humans, and reactions to the playback of emotional voices: (**a**) correlation between the total chronic stress score (TCSS) and the number of scans with ears backwards after the broadcast of the fearful voice in the overall population, (**b**) correlation between TCSS and the number of vigilance/alarm behaviors after the playback of the fearful voice in RC horses; (**c**) correlation between TCSS and the latency of reaction (head turning) in response to the fearful voices in RC horses; (**d**) number of negative behaviors towards humans in the human–horse relationship tests and the number of vigilance/alarm behaviors after the broadcast of the fearful voice in RC horses. Spearman correlations. *: *p* < 0.05; **: *p* < 0.01.

At the overall population level, data analysis showed a significant positive correlation between the total chronic stress score (TCSS) and the time the horses spent with their ears backwards in response to fearful voices (N = 23; r = 0.550; *p* = 0.007; Spearman correlations) (Figure 8a). TCSS was also negatively correlated with the number of frustration behaviors after the playback of sad voices (N= 24; r = −504; *p* = 0.012, Spearman correlations).

As expected, the two populations showed significant differences in their welfare state: RC horses registered a higher TCSS than NRC horses (N = 26; U = 160.00; *p* = 0.000, Mann–Whitney test), which indicates a better welfare state in the NRC horses. We found no statistically significant correlations between any of the welfare measures and the behavioral responses to all the emotional playback categories for the Non-Restricted Conditions horses (NRC) (*p* > 0.05; Spearman correlation). On the contrary, in RC horses, which had more compromised welfare, clear relationships appeared between their welfare state and their reactions to the emotional human voices. Thus, TCSS was both positively correlated with vigilance/alarm behaviors in response to the fearful voice (N = 12; r = 0.706; *p* = 0.010; Spearman correlation) (Figure 8b) and negatively correlated with the latency of reaction (N = 11; r = −0.607; *p* = 0.048, Spearman correlation) (Figure 8c), indicating that horses with poorer welfare reacted faster by turning their heads and showed a significantly increased alarm state in response to fearful voices. A negative correlation between TCSS and AAC (i.e., values lower than the baseline) was also observed in response to happiness in RC, suggesting that the poorer the welfare, the lower the AAC values (N= 10; r = −0.672; *p* = 0.033; Spearman correlation), which describe an increase in the horses’ heart rate. Moreover, a positive correlation was found between TCSS and the time spent gazing at the loudspeaker for happiness, indicating that the poorer the welfare state, the higher the time the animals spent gazing at the loudspeaker during the happiness playback (N = 11; r = 0.722; *p* = 0.012, Spearman correlation). Finally, the number of stereotypic behaviors/hour observed in the home environment was positively correlated with both the number of frustration behaviors after the playback of the happy voice (N = 12; r = 0.592, *p* = 0.043; Spearman correlation) and the number of vigilance/alarm behaviors after the playback of the fearful voice (N = 12; r = 0.594, *p* = 0.042; Spearman correlation).

These data reveal clear correlations between welfare state and perception of emotional human voices. Horses in a good welfare state appear to show little sensitivity to the emotional content of human voices, whereas horses with the poorest welfare showed increased negative reactions to the fearful voice but also to the happy voice. These horses exhibited faster reactions, more alarm behaviors, and increased heart rates. This further differentiated both populations studied, as NRC horses were in a better welfare state than the RC horses overall.

### 3.5. Horses’ Relationships with Humans Influence Their Responses to Emotional Voices

At the overall population level, the number of positive behaviors toward the experimenter during the Human–Horse Relationship (HHR) tests was positively correlated with the time horses spent with their ears forwards in response to the happy voice (N = 23; r = 0.429; *p* = 0.041; Spearman correlation), as well as with the expression of frustration-related behaviors in response to the sad voice (N = 22; r = 0.481; *p* = 0.023; Spearman correlation). Conversely, the number of negative behaviors toward the experimenter during the HHR tests was positively correlated with the number of visual attention behaviors in response to sadness (N = 24; r = 0.443; *p* = 0.030; Spearman correlations).

Significant differences were observed between the two horse populations in their relationships with humans, as indicated by the HHR test: NRC horses exhibited more positive behaviors than RC horses (N = 24; U = 9.5; *p* = 0.000; Mann–Whitney test), while RC horses displayed more negative behaviors (N = 26; U = 145.50; *p* = 0.000, Mann–Whitney test).

When examining the responsiveness of the two populations to emotional stimuli separately, we found that, in the NRC population, there was no correlation between behaviors toward the experimenter in the HHR test and the horses’ responses to emotional voices (*p* > 0.05 Spearman correlation). This lack of correlation may be due to the generally positive attitudes of all NRC horses toward humans.

In contrast, in RC horses, a positive correlation was observed between positive behaviors toward humans and the latency of the head-turning response to the fearful voice (N = 9; r = 0.668; *p* = 0.049, Spearman correlations), suggesting that horses with more positive relationships with humans exhibited a longer latency to respond to the fearful voice. Additionally, positive behaviors toward humans during the HHR test were negatively correlated with the area under the curve (AUC) values in response to the angry voice (N = 7; r = −0.874; *p* = 0.010, Spearman correlations), indicating that the higher the positive attitude toward humans, the lower the increase in the cardiac activity in response to the angry voice. Furthermore, negative behaviors toward humans during the HHR tests were negatively correlated with the latency of the reaction to the fearful voice (N = 11; r = −0.627; *p* = 0.039, Spearman correlations) and positively correlated with the number of vigilance/alarm behaviors exhibited in response to the fearful voice (N = 12; r = 0.637; *p* = 0.026; Spearman correlation) (Figure 8d). This suggests that the more negative the relationship with humans was, the faster the reaction and the higher the vigilance/alarm levels elicited by the fearful voice.

These results show that the perception horses have of humans greatly influences the way horses react to human voices. Horses with a good relationship with humans showed more positive reactions to the happy voice and more visual attention for sadness. The HHR clearly differed between both populations, with all NRC horses showing almost only positive behaviors towards the experimenter contrarily to the RC horses, which most often reacted negatively in the tests. RC horses, however, differed individually, and it appeared that the poorer the HHR, the more rapidly and strongly (alarm) they reacted to the fearful voice and the higher their heart rate increased in response to the angry voice. Thus, the better the relationship with humans, the less emotional voices induced different emotional reactions, and the poorer this human–horse relationship, the more horses perceived negative-emotional human voices as a potential threat.

## 4. Discussion

The results of this study reveal that horses’ reactions to emotional human voices were highly influenced by their affective states and earlier experiences with humans. Behavioral, cardiac, and brain responses converged to show that horses experiencing different life and riding conditions reacted in very different ways to the different voices, but also that, within the same population, individual responses were highly modulated by individuals’ affective states and perception of humans. Horses discriminated between the different voices, but the significance for the horses tested may have differed.

*An attentional bias for negative voices: recognition, empathy, experience, or universal coding?*

At the overall populational level, we observed more head turns for all the negative voices, whatever their intensity, than expected by chance, which was not the case for the positive voice. One limitation, of course, is that we had only one voice with a positive valence and three with negative valences. However, we did not find any other voice stimulus where the positive valence would not be ambiguous. Given our results showing differences in horses’ responses to fearful, angry, and sad voices, comparing this sole positive voice to only one of the possible negative voices, as in Smith et al. [9], would have given a limited view of emotional processing. Furthermore, Maigrot et al. [2] also found that Equidae and Suidae reacted more to human negative than positive voices, but only when these voices were first broadcasted. Smith et al. [9] found more time in vigilant (i.e., alarm [31]) postures in response to an angry than a happy voice in domestic horses, and Siniscalchi et al. [7] found that happiness was not processed the same way as all negative human voices broadcasted to dogs. After the playback of an angry human voice, cats express more stress behaviors [8], dogs stop eating [7] and horses spend more time in vigilant posture and have a higher heart rate than after that of a positive voice [11]. Auditory information seems to prevail over visual information, as there is no discrimination between emotional faces if a neutral sound is broadcast in experiments testing cross-modal recognition of human emotions (horses: [11], dogs: [53], cats: [8]). Studies have, overall, suggested a cross-taxa universal coding of emotions, possibly based on a universal relationship between the structure of vocalizations and their emotional content [6,27]. However, in a study on human perception of human voices and those of other species with varying degrees of phylogenetic relatedness, subjects recognized the negative (agonistic) voices of dogs and chimpanzees but were unable to recognize the emotional content of any of the calls of an unknown species (tree shrew) [27]. The authors concluded that there is little evidence of evolutionarily retained mechanisms for cross-taxa emotional recognition from voice and that experience is necessary for emotion recognition, which is the case, to varying degrees, for most species used for such testing (e.g., cats: [54]; dogs: [55]; pigs: [56]), including chimpanzees, which they may encounter in zoos or through media [27].

Taken together, these findings suggest two complementary interpretations that may help contextualize our results: the heightened responsiveness to negative human voices across species likely reflects their greater salience and adaptive relevance, as supported by several studies; however, recent work has also emphasized the role of experience and phylogenetic relatedness in the recognition of emotional content across species, challenging the idea of a universally retained cross-taxa coding system. These findings clearly rule out the possibility of a mere perceptual processing of acoustic structures.

*Evidence of experience-dependent processing of the emotional content of voices: differences between populations.*

In our study, whereas the Non-Restricted Conditions horses (NRC) reacted most to the angry voice by turning their head and spending more time with their ears backwards, the Restricted Conditions horses (RC) showed a higher heart rate for all the negative voices than for the positive voice. They were more attentive and spent more time with their ears backwards in response to the fearful and sad voices than the NRC horses and reacted more quickly to the angry voice. There are few examples of variations between populations in the processing of emotional voices. Most studies performed on reactions to emotional human voices have either used animals living in one controlled environment (e.g., horses: [11]) or owned pet animals (e.g., cats: [8], dogs: [7]) living in a variety of households. Also, their different origins are not often taken into account during the analyses (e.g., horses: [9]). In their study on goats, Rosenberger et al. [15] found large variations in cognitive performance according to the site where they were living and being tested, despite having the same genetic origins and being tested by the same experimenters. Barber et al. [14] found large differences in visual attention (eye-tracking) when facing emotional human faces between pet dogs and lab dogs (living in groups in enclosures with limited contact with humans). In particular, the lab dogs looked longer at the negative face than the positive one, whereas the reverse was observed for the pet dogs. The authors suggested that the difference in the amount of contact with humans may be responsible for these differences, but also the quality of the human–animal interactions may have differed, as well as their welfare states. Horses living in restricted conditions appeared to be more sensitive to the valence of past experiences associated with a human voice than horses living in more naturalistic conditions, showing more forward ears (and relaxed posture) for the voice associated with earlier positive interactions and more frustration for the voices associated with earlier negative experience [18].

Our results, in line with previous findings, suggest that the processing of emotional human voices in animals may be shaped by their living conditions and experience with humans, underlining the importance of environmental context in emotional voice perception.

*Welfare, affective state, and perception biases of auditory emotional content.*

The two populations we tested showed clear opposite trends in terms of welfare. Horses from the population with more compromised welfare (RC horses) showed a clear bias in their responses (increased heart rate) to the negative voices, and still more so for those that had the highest score for total chronic stress. Overall, we found enhanced responsiveness to negative emotional voices for the horses that were in the most compromised welfare states within the same populations. An earlier study, performed on the same types of populations, showed that horses living in more restricted conditions had a more “pessimistic” perception of ambiguous stimuli [39]. Anxious people bias their attention towards threatening stimuli [57]. It is also well known that people suffering from affective disorders such as depression are more prone to preferentially processing negative stimuli [22]. Depressed/apathetic horses tend to react more strongly to novel objects in a familiar environment than “control” horses [58]. Affective states result from the interaction between discrete emotions, such as short-term responses, and a long-term background mood [4], and can modulate individuals’ attention and judgment [1,23]. Animals’ moods differ as a result of their housing and husbandry environment [59]. Improvements in life conditions (including the human–animal relationship) have a clear positive impact on the moods of animals and their cognitive processing of sensory stimuli (e.g., [24,25]). Reefmann et al. [16] found that sheep living in an “impoverished” group had more reactions to negative human voices, but also an increased sensitivity to valence (more negative reactions to negative voices, more positive to positive voices; see also [18] for horses). The question remains of whether a positive mood is stabilizing, or whether there is an increased excitability in a more negative mood [16]. Moreover, it is not clear what part may be due to the life conditions and what part may be due to the quality of the human–animal relationship. Taken together, these findings suggest that compromised welfare and negative affective states could have biased the animals’ perception toward negative emotional stimuli, highlighting the influence of mood and living conditions on the cognitive processing of human vocal emotions.

*Human–animal relationship and the perception of the emotional content in human voice.*

Human–animal relationships are built upon the repetition of interactions, in which valence is memorized, and their quality is reflected in the reactions of individuals in human–animal relationship tests [60]. In the present study, all our NRC horses had a mostly positive perception of humans, as shown by their reactions in the HHR tests, which may easily be explained by their more relaxed context of interaction with humans and the predominant use of positive reinforcement during their training to accept care or being ridden (e.g., [61]). Within the RC population, the more positive horses were towards the experimenter, the slower their responses and the lower their heart rate increased after the playback of the angry voice; they appeared less affected emotionally than the horses that showed a negative relationship with humans. Reversely, the more negative their behavior towards the experimenter during the HHR test, the more they reacted after the playback of the fearful voice (shorter latencies and more vigilance/alarm). Horses do generalize their perception of familiar people to unfamiliar people (e.g., [19]), which means that if their “routine” interactions with their caretakers are not positive, they may see humans, in general, as a threat. Several studies have shown that the perception of emotional cues in the voice can be influenced by familiarity. Thus, horses discriminated between congruent/incongruent visual/auditory human emotional cues only if they were from a familiar human, as shown in Nakamura et al. [13] study. Captive western gorillas react three times more to the voice of a familiar human with whom they have had negative interactions than to that of an unfamiliar human [17]. There is a positive correlation between their score of attachment to their owner and dogs’ neural activity increases [62]. The ability of horses to recognize individual human voices is influenced by the valence of past interactions [18]. Mason et al. [63], in their study of goats, found limited evidence of discrimination between human emotional cues. They suggested that past experiences with human voices—especially those linked to either positive or negative events—could affect how well animals respond to these cues. In our study, the RC horses interacted with a larger and more varied group of humans, including riding instructors, caretakers, and a range of regular riding learners. This likely exposed them to a wider array of human emotional states, including fear or anger, and their vocal expression and subsequent possible associated actions. For example, beginner riders, who might express fear, could unintentionally cause stress to the horse through actions like tightening the reins, which could also be uncomfortable or even painful for the animal [64]. In contrast, the NRC horses were more like family pets, interacting with a smaller, more familiar group of handlers in a calmer environment and being ridden by experienced riders for leisure activities in a relaxed way [64]. There was therefore less chance that humans expressed fear or anger during their encounters with the horses. While we did not measure the specific types of interactions or how much the horses were exposed to genuine human emotional states, these differences, also reflected in the results of the human–horse relationship tests, may help explain the results we observed. Therefore, future studies on how domestic animals perceive human emotions should take into account the nature of their interactions with humans [65].

*How it all works: brain processing of auditory emotional content.*

Lateralization of emotional processing in animals has been studied either indirectly through behavioral responses or, more rarely, by directly investigating brain processing [1,66,67]. We used both approaches and looked both at EEG responses and head turns in response to the playback, as it is assumed that the perception of a stimulus with the right eye indicates left hemisphere processing and vice versa [68]. Actually, we found almost no laterality bias in head-turning responses, apart from right head-turning for the fearful voice in NRC horses, which was surprising, as most studies on the reactions of animals to human voices have found lateralized head turns [7]. However, Barber et al. [14] found no difference in laterality according to the valence of the emotional human voice in dogs. Familiarity with the voice may be needed in order to trigger a lateralized behavioral response, as we found a right head turn for voices associated with earlier positive interactions with humans (and none for voices associated with negative experiences) in an earlier study [18]. In the present case, right head turns in response to fearful voices were observed in NRC horses, which may have had less experience with intense human emotional expressions, which may reflect a left hemisphere involvement in the cognitive evaluation of the emotional cue. Similar patterns were observed in dogs, where left nostril (left hemisphere) use was reported in response to human fear odors [69], possibly indicating the involvement of the left amygdala in more refined discrimination of emotional cues. This suggests that NRC horses may have engaged in a more detailed evaluative process of an unusual or unexpected sound, a phenomenon also observed when horses hear whinnies from non-group members [47].

Lateralization patterns appeared much more clearly in the EEG recordings. These rapid, more lateralized responses at the brain level as compared to the behavioral responses may reflect a two-step process in cognitive processing, as already proposed by d’Ingeo et al. [18] and suggested by Basile et al. [47], who found that, in response to conspecific vocal stimuli, horses could have different lateralized patterns between immediate ear turns (Pryer reflex) and later head turns, especially for non-familiar voices. Brain responses to sensory stimuli—particularly those linked to negative emotions—are very rapid, with early perceptual processing occurring before more elaborate cognitive evaluation [70]. This temporal delay between initial and higher-order processing may help further explain why lateralization patterns are more clearly observed at the neural level than in overt behavioral responses.

There were clear opposite trends between NRC and RC horses in EEG lateralization. In NRC horses, gamma wave activity increased in the right hemisphere (RH) after hearing happy voices, while in RC horses, it decreased after angry voices. No notable EEG changes were observed in the left hemisphere (LH) of RC horses in response to happy voices. Both groups showed a decrease in LH beta waves after sad voices, but only NRC horses exhibited increased theta activity, suggesting a different attentional response. RC horses showed a bilateral theta increase in response to anger and fear, supporting an attentional bias toward negative emotions and confirming lateralized processing of emotional stimuli, whereas no such changes were observed in NRC horses.

The increase in RH gamma in NRC horses likely reflects heightened arousal, consistent with studies linking RH activity to attention toward arousing stimuli (e.g., [1,7,38,68]). Gamma has been associated with positive stimuli in horses [18,71], and with attention toward neutral stimuli or expectancy in humans [36,46]. Positive reinforcement during training is also known to enhance attention in horses [38]. Although the LH has been linked to positive emotion processing [68], the observed beta decrease and theta increase in NRC horses in response to sadness—a low-arousal negative emotion—may reflect more complex attentional demands for an emotion possibly more difficult to “interpret” for the horse.

Anger and fear did not elicit EEG asymmetries but did trigger lateralized behaviors, such as right head-turning for fear, and ear-back postures for anger, indicating negative perception. These findings are consistent with our previous study on horses’ perception of emotional acoustic stimuli [18], where an increased proportion of gamma waves in the right hemisphere was observed in response to a human voice associated with a prior positive experience, while no lateralization was detected for negatively associated voices. In RC horses, the bilateral theta increase likely reflects cognitive processing of high-arousal negative stimuli, as theta activity has been linked to differentiation of emotional versus neutral stimuli and arousal-based processing [72,73]. Although our scalp EEG lacks spatial resolution, prior research suggests theta activity relates to memory processing and emotional perception in the frontocortical and limbic regions [73]. Theta is also associated with increased attention and behavioral readiness [73] and is influenced by individual traits such as anxiety [74]. It may reflect internal demands involving memory coordination [75] and hippocampal encoding, though direct involvement cannot be confirmed due to the high discrepancy between the method used in that study (intracranial electrodes that give precise information on the locations), and the present study. However, this information brings some insights on the correlations between theta activity and emotional perception that provide useful hints for interpreting some of our results at the scalp level.

The pronounced theta increase in RC horses in response to anger and fear may indicate strong fear reactivity and corresponds with observed vigilance/alarm behaviors. Theta has been linked to both active movement and immobility in response to threats (e.g., freezing) [37,76], which aligns with the alarm postures seen in RC horses. Although hippocampal involvement cannot be confirmed via scalp EEG, the convergence of theta activity and behavior warrants further investigation.

RC horses may have more frequent exposure to human anger and fear in riding centers than NRC horses, leading to associative memories. This could be related both to their encounters with a larger number and diversity of humans but also to the higher chance that fear and anger are expressed in the context of riding lessons than relaxed outdoor riding. Horses are sensitive to olfactory and postural fear signals in humans [77], which may become linked with vocal cues. The absence of strong EEG responses to high-intensity negative voices in NRC horses likely reflects their positive welfare and human relationships. The sad voice, being low in arousal, may be more familiar, as NRC caretakers express less high negative states but may occasionally express sadness, which potentially explains the horses’ observed theta response. Overall, these findings challenge the “valence theory” [1]—suggesting LH processing of positive stimuli and RH for negative and arousing stimuli—and highlight the need for further studies combining behavioral and neural measures of lateralization in response to emotional valence and arousal, taking into account the influence of exposure and life conditions on the perception of emotion in domestic animals.

## 5. Conclusions

This study, which tested the behavioral, cardiac, and brain responses of horses to the playback of emotional human voices, clearly reveals that there are cognitive biases in how horses perceive emotional cues in human voices. It provides further evidence that welfare state and relationships with humans influence how horses “see the world”, including what valence they give to human emotional cues. These findings are an important contribution to the debates on cross-taxa perception and recognition of acoustic emotional cues, clearly demonstrating that transmission of emotional content is not as straightforward nor as clear-cut as generally assumed. In a more applied direction, these results are also of the highest importance, as they show that while “happy horses” may not be worried by expressions of negative emotions by humans, horses in a negative affective state may, on the contrary, overlook positive cues in humans and be oversensitive to any sign of human negative emotion. In both cases, there may be a discrepancy between horses’ perceptions and humans’ emotional states that may lead to inappropriate interactions. The study also emphasizes the potential of studying EEG power spectrum for animals as a way of achieving more insight into the cognitive processing of emotional cues. For these different reasons, this study may pave the way for an enlarged and deeper view on the decoding of cross-taxa emotional cues.

## Data Availability

The dataset analyzed in the current study is included in this published article (as its Supplementary Materials).

## Supplementary Materials

The following supporting information can be downloaded at: https://www.mdpi.com/article/10.3390/ani15213217/s1, Figure S1: Examples of acoustic stimuli presented to the horses; Figure S2: Examples of “horse type” and “pony type” included in the EEG analysis; Table S1: Details of the horses included in the experiment; Table S2: Ethogram used for the behavioral analysis; Table S3: Descriptive statistic of behavioral parameters for the overall population; Table S4: Descriptive statistic of behavioral parameters for the Non-Restricted Conditions horses; Table S5: Descriptive statistic of behavioral parameters for the Restricted Conditions horses; Table S6: Descriptive statistics of EEG data.
