# Supplementary material for: Chronic State and Relationship to Humans Influence How Horses Decode Emotions in Human Voices: A Brain and Behavior Study
Source: Animals (Basel). 2025 Nov 5;15(21):3217. doi: 10.3390/ani15213217 (PMC12610170; doi:10.3390/ani15213217)
Supplement: Supplementary file 1 [file animals-15-03217-s001.zip › Supplementary files/SI_d'Ingeo et al..pdf]

Chronic state and relationship to humans influence how horses decode emotions in human voices: a brain and behavior study

Serenella d'Ingeo[1,2], Marcello Siniscalchi[1], Angelo Quaranta[1], Hugo Cousillas[2†], and Martine Hausberger[3,4†]

1 Department of Veterinary Medicine, Animal Physiology and Behavior Research Unit, University of Bari Al-do Moro, 70121 Bari, Italy;
2 Laboratoire Ethologie Animale et Humaine -EthoS, UMR 6552 –CNRS; University of Rennes, University of Caen-Normandie, -,35 042 Rennes Cedex, France
3 Integrative Neuroscience and Cognition Center, UMR 8002-CNRS, University of Paris-Cité, 75006 Paris, France
4 Honorary Professor, Department of Entomology and Zoology, Rhodes University, 6190 Makhanda, South Africa
† These authors contributed equally

Corresponding authors: Serenella d'Ingeo
Email:serenella.dingeo@uniba.it

Table S1. Details of the horses included in the experiment. RC: Restricted Conditions; NRC: Non-Restricted Conditions.

Horse	Breed	Group	Type	Sex	Age (years)
1	Thoroughbred	RC	Horse	Mare	9
2	Unregistered	RC	Horse	Gelding	8
3	Unregistered	RC	Pony	Gelding	15
4	Unregistered	RC	Pony	Mare	17
5	French Pony	RC	Pony	Mare	9
6	French Pony	RC	Pony	Gelding	12
7	French saddlebred	RC	Horse	Mare	15
8	Thouroughbred	RC	Horse	Mare	13
9	Unregistered	RC	Horse	Mare	11
10	Unregistered	RC	Horse	Gelding	10
11	Angloarab	RC	Horse	Gelding	12
12	French Pony	RC	Pony	Mare	11
13	Unregistered	RC	Horse	Gelding	5
14	Unregistered	NRC	Pony	Stallion	6
15	Unregistered	NRC	Pony	Gelding	13
16	Unregistered	NRC	Pony	Gelding	22
17	Unregistered	NRC	Horse	Mare	20
18	Unregistered	NRC	Pony	Mare	13
19	ThouroughbredxFriesan	NRC	Horse	Mare	16
20	French saddlebred x Friesan	NRC	Horse	Mare	4
21	Unregistered	NRC	Pony	Stallion	5
22	Unregistered	NRC	Pony	Stallion	4
23	French Pony	NRC	Pony	Mare	5
24	Unregistered	NRC	Pony	Mare	2
25	Unregistered	NRC	Horse	Mare	3
26	Unregistered	NRC	Pony	Mare	4
27	Unregistered	NRC	Horse	Mare	19

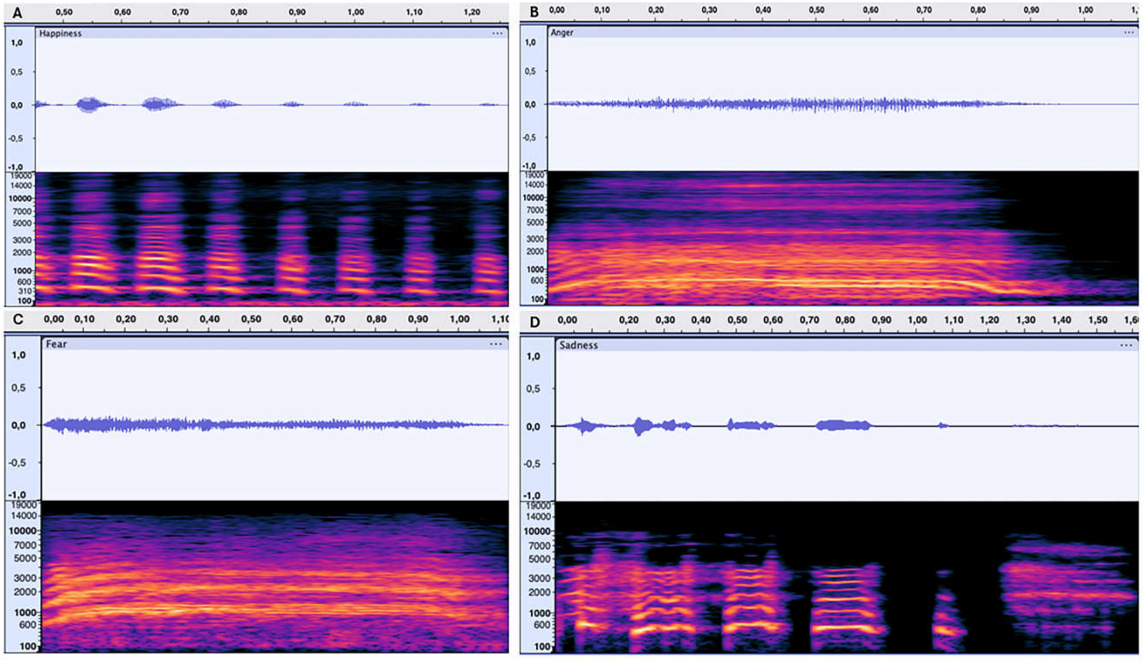

Figure S1. Examples of acoustic stimuli presented to the horses. Emotional voices of A) Happiness, B) Anger, C) Fear and D) Sadness. x-axis: time; y-axis: amplitude (on a scale from -1,0 to + 1,0) and Hz (100-1900).

Table S2. Ethogram used for the behavioral analysis [21,49].

**Behavioural categories**	**Description**
**Vigilance/Alarm**	The horse remains immobile on all four legs without shifting position. The head and neck are held upright, always above the level of the withers. Eyes are wide open, focused, and directed toward a stimulus. The base of the tail is often elevated above the dorsal line. Nostrils can be dilated.
**Frustration**	The horse exhibits repetitive chewing motions performed without the presence of food or oral stimuli (vacuum chewing); move the upper lip vertically without contact to any object, often with repetitive smacking by bringing the lips together and apart. The forelimb could be used repetitively to strike or scrape the ground (pawing). The mouth could be opened widely with deep inhalation during yawning. The horse uses a limb or hoof to rub or scrape parts of its body (self-scratching). The horse may also turn away from the stimulus.
**Visual attention directed toward the loudspeaker**	The horse directs its head and eyes toward the loudspeaker. This includes glancing (< 1s) or gazing (> 1s) at the loudspeaker and turning its body in the direction of the loudspeaker.
**Ear position**	*Forward*: Pavilion towards the front, not visible from the side; *Backward*: Pavilions oriented towards the back, visible from back but not from aside; *Sideward*: Pavilions oriented on the animal's sides, pavilions visible from the side but not from front or back.
**Head-turning**	Lateral movement of the head to one side, without movement of the rest of the body.

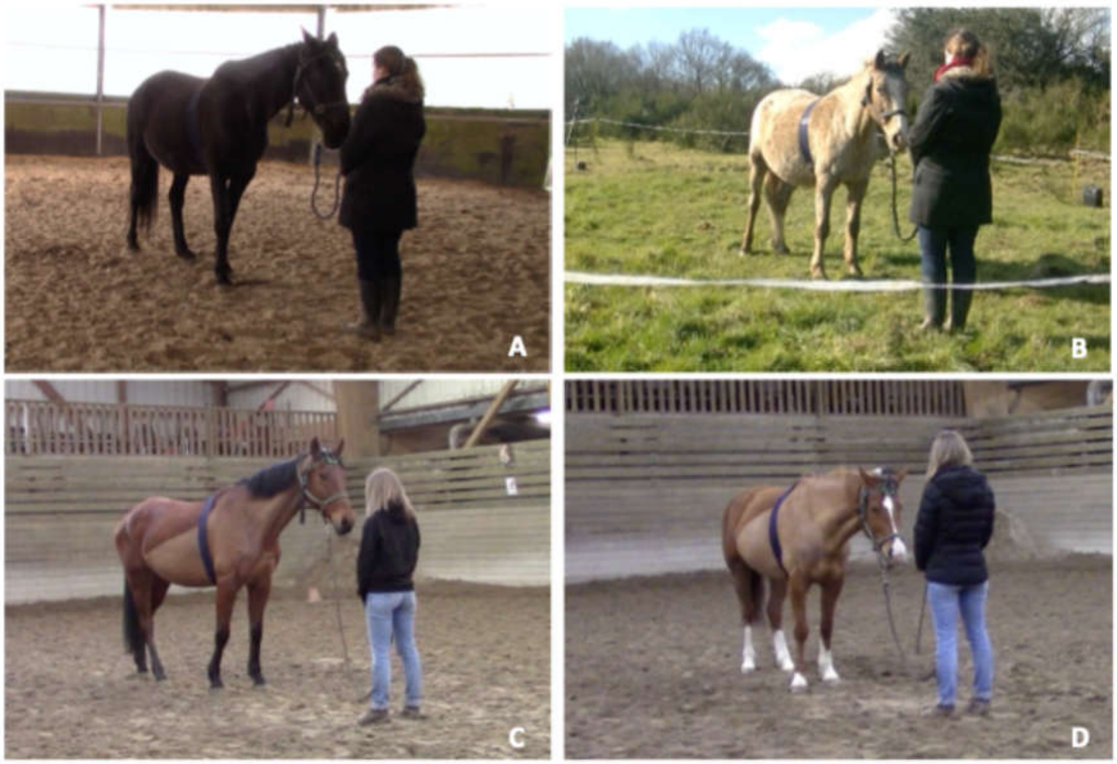

Figure S2. Examples of "horse type" and "pony type" included in the EEG analysis. (A) Horse type under Non-Restricted Conditions; (B) Pony type under Non-Restricted Conditions; (C) Horse type under Restricted Conditions; (D) Pony type under Restricted Conditions.

Table S3. Descriptive statistic of behavioral parameters for the overall population.

Behavioural parameter	Emotion	N	Mean	Standard Deviation
Latency	Happiness	23	3.18	2.25
	Sadness	26	2.20	1.93
	Anger	23	2.49	1.80
	Fear	24	2.67	1.94
Number head turning	Happiness	26	0.58	0.50
	Sadness	26	0.85	0.37
	Anger	23	0.91	0.29
	Fear	24	0.79	0.88
Vigilance	Happiness	26	0.85	1.22
	Sadness	25	1.44	1.23
	Anger	26	1.31	1.23
	Fear	25	1.76	1.59
Frustration	Happiness	25	0.40	0.71
	Sadness	24	0.29	0.62
	Anger	26	0.73	0.83
	Fear	24	0.54	0.83
Visual attention loudspeaker	Happiness	25	2.44	1.23
	Sadness	24	2.83	0.92
	Anger	24	2.13	1.15
	Fear	24	2.67	1.24
Ears forward	Happiness	25	1.92	1.68
	Sadness	26	1.46	1.45
	Anger	26	1.69	1.54
	Fear	24	1.88	1.36
Ears backward	Happiness	26	2.62	1.92
	Sadness	25	2.56	1.69
	Anger	26	3.19	1.50
	Fear	23	2.00	1.04
Ears asymmetric	Happiness	26	1.23	1.18
	Sadness	26	1.77	1.63
	Anger	25	0.84	1.14
	Fear	25	1.40	1.41
Time gazing loudspeaker	Happiness	22	7.22	5.14
	Sadness	26	8.86	6.61

	Anger	25	9.32	7.47
	Fear	23	7.92	5.44
AUC	Happiness	10	27149.70	30070.82
	Sadness	9	27671.89	26225.79
	Anger	9	34680.67	22969.54
	Fear	12	38011.75	28060.18
AAC	Happiness	10	11397.90	15540.94
	Sadness	8	2256.00	4084.69
	Anger	8	1833.63	3611.16
	Fear	11	6538.55	10857.00
TCSS		26	7.27	5.30
Positive behaviors (HHRT)		24	6.17	4.76
Negative Behaviours (HHRT)		26	2.15	4.09
Stereotypies		13	7.92	10.19

Table S4. Descriptive statistic of behavioral parameters for the Non-Restricted Conditions horses.

Behavioural parameter	Emotion	N	Mean	Standard Deviation
Latency	Happiness	11	2.60	1.96
	Sadness	13	2.57	2.08
	Anger	11	3.25	1.95
	Fear	13	3.05	2.16
Number head turning	Happiness	13	0.69	0.48
	Sadness	13	0.77	0.44
	Anger	11	0.91	0.30
	Fear	13	0.69	0.48
Vigilance	Happiness	13	0.85	1.41
	Sadness	12	0.83	0.94
	Anger	13	0.92	1.19
	Fear	13	1.39	1.66

Frustration	Happiness	13	0.54	0.78
	Sadness	12	0.58	0.79
	Anger	13	1.00	0.91
	Fear	13	0.62	0.65
Visual attention loudspeaker	Happiness	12	2.50	1.17
	Sadness	13	2.46	0.78
	Anger	13	1.85	1.14
	Fear	12	2.08	1.00
Ears forward	Happiness	13	2.31	1.60
	Sadness	13	1.62	1.66
	Anger	13	1.69	1.65
	Fear	13	1.69	1.49
Ears backward	Happiness	13	1.92	1.80
	Sadness	12	1.75	1.14
	Anger	13	3.15	1.63
	Fear	12	1.42	0.90
Ears asymmetric	Happiness	13	1.69	1.32
	Sadness	13	2.23	1.59
	Anger	13	0.92	1.12
	Fear	13	2.00	1.63
Time gazing loudspeaker	Happiness	11	8.07	5.11
	Sadness	13	7.12	4.11
	Anger	12	7.28	8.25
	Fear	11	6.43	6.19
TCSS		13	3.31	0.63
Positive behaviors (HHRT)		13	9.00	4.74
Negative Behaviours (HHRT)		13	0.15	0.55
Stereotypies		0	0.00	0.00

Table S5. Descriptive statistic of behavioral parameters for the Restricted Conditions horses.

Behavioural parameter	Emotion	N	Mean	Standard Deviation
Latency	Happiness	12	3.70	2.45
	Sadness	13	1.84	1.79
	Anger	12	1.79	1.39
	Fear	11	2.23	1.64
Number head turning	Happiness	13	0.46	0.51
	Sadness	13	0.92	0.28
	Anger	12	0.92	0.29
	Fear	11	0.91	0.30
Vigilance	Happiness	13	0.85	1.07
	Sadness	13	2.00	1.22
	Anger	13	1.69	1.18
	Fear	12	2.17	1.47
Frustration	Happiness	12	0.25	0.62
	Sadness	12	0.00	0.00
	Anger	13	0.46	0.66
	Fear	11	0.46	1.04
Visual attention loudspeaker	Happiness	13	2.39	1.33
	Sadness	11	3.27	0.90
	Anger	11	2.46	1.13
	Fear	12	3.25	1.22
Ears forward	Happiness	12	1.50	1.73
	Sadness	13	1.31	1.25
	Anger	13	1.69	1.49
	Fear	11	2.09	1.22
Ears backward	Happiness	13	3.31	1.84
	Sadness	13	3.31	1.80
	Anger	13	3.23	1.42
	Fear	11	2.64	0.81
Ears asymmetric	Happiness	13	0.77	0.83
	Sadness	13	1.31	1.60

	Anger	12	0.75	1.22
	Fear	12	0.75	0.75
Time gazing loudspeaker	Happiness	11	6.37	5.26
	Sadness	13	10.61	8.22
	Anger	13	11.20	6.41
	Fear	12	9.29	4.48
AUC	Happiness	10	27149.70	30070.82
	Sadness	9	27671.89	26225.79
	Anger	9	34680.67	22969.54
	Fear	12	38011.75	28060.18
AAC	Happiness	10	11397.90	15540.94
	Sadness	8	2256.00	4084.69
	Anger	8	1833.63	3611.16
	Fear	11	6538.55	10857.00
TCSS		13	11.31	4.80
Positive behaviors (HHRT)		11	2.82	1.54
Negative Behaviours (HHRT)		13	4.15	5.08
Stereotypies		13	7.92	10.19

Table S6. Descriptive statistics of EEG data. Mean and standard deviation are reported for the 2 seconds before stimulus onset and the 2 seconds after. LH: Left Hemisphere; RH: Right Hemisphere; NRC: Non-Restricted Conditions (N= 5); RC: Restricted Conditions (N= 6).

EEG wave type	Population	Emotion	Mean		Standard Deviation	
			Before	After	Before	After
Delta- LH	NRC	Happiness	4.50	1.41	8.05	6.87
		Sadness	0.63	1.30	4.96	3.74
		Anger	5.21	6.58	4.39	6.73
		Fear	0.63	1.73	2.26	4.12
	RC	Happiness	1.68	0.83	2.70	3.10
		Sadness	0.93	2.51	1.90	2.00
		Anger	0.58	3.28	0.73	4.80
		Fear	0.43	2.22	1.22	4.60

Delta- RH	NRC	Happiness	4.14	2.27	3.42	2.37
		Sadness	1.10	2.34	1.47	1.86
		Anger	2.01	3.37	5.76	5.17
		Fear	1.40	1.62	4.72	1.77
	RC	Happiness	1.69	1.36	2.79	1.62
		Sadness	1.27	1.11	1.31	3.13
		Anger	0.62	2.32	2.42	3.16
		Fear	0.78	4.08	1.75	2.49
Theta- LH	NRC	Happiness	27.25	18.82	22.33	31.37
		Sadness	16.09	29.26	21.02	21.89
		Anger	32.08	42.35	27.61	13.40
		Fear	15.78	20.13	7.19	12.75
	RC	Happiness	20.27	17.06	7.38	11.93
		Sadness	14.43	24.12	6.89	13.09
		Anger	10.83	29.28	12.74	16.69
		Fear	6.34	28.29	14.89	18.55
Theta- RH	NRC	Happiness	51.12	36.40	13.84	15.86
		Sadness	13.41	20.20	7.45	10.80
		Anger	52.46	40.89	27.71	33.62
		Fear	19.43	24.17	18.33	21.11
	RC	Happiness	17.59	23.40	20.50	17.77
		Sadness	13.50	21.79	8.61	9.64
		Anger	13.44	23.86	16.08	16.47
		Fear	18.88	25.13	13.93	18.81
Alpha- LH	NRC	Happiness	10.21	8.85	2.06	1.73
		Sadness	10.71	6.19	2.45	2.51
		Anger	5.69	6.61	6.18	4.05
		Fear	6.56	6.39	2.05	2.19
	RC	Happiness	9.08	4.60	1.19	3.58
		Sadness	5.32	6.11	3.24	2.39
		Anger	6.63	5.53	3.33	2.75
		Fear	4.20	3.60	2.33	2.99
Alpha- RH	NRC	Happiness	9.38	5.55	3.32	3.04
		Sadness	7.75	4.90	2.72	1.32
		Anger	9.98	7.91	3.28	2.39
		Fear	9.31	7.77	4.54	3.91
	RC	Happiness	12.27	7.08	4.60	2.60
		Sadness	6.16	6.22	4.76	2.48
		Anger	5.66	7.21	5.60	6.80
		Fear	5.24	8.48	1.91	2.01
Beta- LH	NRC	Happiness	32.75	34.42	16.61	17.20
		Sadness	40.55	33.10	14.30	11.04
		Anger	28.97	23.25	14.94	10.27

		Fear	36.96	35.37	6.12	8.23
	RC	Happiness	26.92	34.07	7.89	6.31
		Sadness	40.90	29.46	4.76	6.04
		Anger	40.78	26.85	7.83	10.96
		Fear	39.00	32.34	5.55	12.54
Beta- RH	NRC	Happiness	23.37	26.27	9.42	6.79
		Sadness	39.84	29.43	4.78	6.29
		Anger	20.31	22.76	17.28	17.14
		Fear	32.96	30.67	14.53	9.36
	RC	Happiness	35.82	33.75	12.08	9.81
		Sadness	38.77	35.88	4.11	6.04
		Anger	39.52	33.80	11.07	9.79
		Fear	39.77	28.18	6.39	9.56
Gamma- LH	NRC	Happiness	22.97	32.46	12.97	15.64
		Sadness	30.95	29.87	11.19	13.34
		Anger	16.32	14.57	16.50	10.17
		Fear	33.83	29.27	9.69	8.80
	RC	Happiness	32.95	33.84	3.08	8.75
		Sadness	39.55	33.43	8.58	6.90
		Anger	40.17	26.91	10.84	10.57
		Fear	48.75	30.03	11.63	9.71
Gamma- RH	NRC	Happiness	13.80	27.56	8.30	8.91
		Sadness	34.21	31.21	8.04	7.66
		Anger	11.21	16.05	15.57	17.63
		Fear	25.07	29.27	13.87	15.13
	RC	Happiness	30.50	31.16	11.00	7.30
		Sadness	36.97	32.97	9.94	8.16
		Anger	36.99	27.27	10.29	9.11
		Fear	33.72	30.75	9.57	12.74
